# NR5A2 Regulates *Lhb* and *Fshb* Transcription in Gonadotrope-Like Cells *In Vitro*, but Is Dispensable for Gonadotropin Synthesis and Fertility *In Vivo*

**DOI:** 10.1371/journal.pone.0059058

**Published:** 2013-03-11

**Authors:** Jérôme Fortin, Vikas Kumar, Xiang Zhou, Ying Wang, Johan Auwerx, Kristina Schoonjans, Ulrich Boehm, Derek Boerboom, Daniel J. Bernard

**Affiliations:** 1 Department of Pharmacology and Therapeutics, McGill University, Montréal, Québec, Canada; 2 Département de Biomédecine Vétérinaire, Université de Montréal, Ste-Hyacinthe, Québec, Canada; 3 Laboratory of Integrative and Systems Physiology, School of Life Sciences, Ecole Polytechnique Fédérale de Lausanne, Lausanne, Suisse; 4 Department of Pharmacology and Toxicology, University of Saarland School of Medicine, Homburg, Saarland, Germany; Baylor college of Medicine, United States of America

## Abstract

Successful mammalian reproduction depends on proper synthesis of the pituitary-derived glycoprotein hormones, luteinizing hormone (LH) and follicle-stimulating hormone (FSH). Several transcription factors cooperate to activate cell-specific and hormone-regulated expression of the gonadotropin beta subunits (*Lhb* and *Fshb*). Among these, NR5A1 (steroidogenic factor 1; SF1) has been shown to directly bind to the *Lhb* promoter, mediate basal and gonadotropin-releasing hormone (GnRH)-stimulated *Lhb* transcription, and possibly directly regulate *Fshb* expression. Recently, the closely-related NR5A2 was shown to activate the rat *Lhb* promoter *in vitro*. Here, we further characterized the role of NR5A2 in regulating gonadotropin synthesis. Ectopically expressed NR5A2 directly activated the murine *Lhb* promoter in a manner identical to that of NR5A1, whereas neither factor activated the murine *Fshb* promoter. In LβT2 gonadotrope-like cells, depletion of endogenous NR5A1 or NR5A2 impaired basal and GnRH-stimulated *Lhb* and *Fshb* transcription. To analyze the physiological role of NR5A2 in gonadotropes *in vivo*, we generated mice with a gonadotrope-specific deletion of *Nr5a2*. In contrast with our *in vitro* data, these mice had normal pituitary *Lhb* and *Fshb* expression and intact fertility. Together, our data establish that NR5A2 can act in a non-redundant manner to regulate *Lhb* and *Fshb* transcription *in vitro*, but is dispensable *in vivo*.

## Introduction

The pituitary-derived gonadotropins, luteinizing hormone (LH) and follicle-stimulating hormone (FSH), are critical regulators of gonadal function and fertility in mammals. LH and FSH are dimeric glycoproteins composed of a common α subunit (αGSU or CGA) and unique β subunits (LHB and FSHB), which confer biological specificity. *Lhb* and *Fshb* expression, which is rate-limiting in the production of the mature hormones, is under the control of several endocrine, paracrine, and autocrine factors [1], [2]. Most important for *Lhb* production is gonadotropin-releasing hormone (GnRH). GnRH is released in a pulsatile manner by a small set of neurons within the preoptic area and mediobasal hypothalamus, binds the GnRH receptor (GnRHR) on gonadotrope cells of the anterior pituitary gland, and potently stimulates LH release and synthesis [3], [4]. LH in turn regulates steroidogenesis by the testes and ovaries, and is critical for ovulation and luteinization in females [5], [6].

Multiple factors control the cell-specific and hormone-induced expression of *Lhb* and *Fshb*. GnRH regulation of *Lhb* is mediated primarily through extracellular regulated kinases 1 and 2 (ERK1/2)-dependent induction of early-growth response 1 (EGR1) [7], [8]. EGR1 then acts in concert with NR5A1 (also known as steroidogenic factor 1; SF1) and *paired-*like homeodomain transcription factor (PITX) proteins at conserved *cis*-elements within the proximal *Lhb* promoter to activate transcription (reviewed in [2]). How GnRH stimulates expression of *Fshb* is less well understood and may differ between species [1]. NR5A1's central role in gonadotrope function is perhaps most clearly demonstrated in mice with pituitary-specific deletion of *Nr5a1* [9], which display hypogonadotropic hypogonadism, with undetectable basal expression of *Lhb*, *Fshb*, and *Gnrhr*. Studies in cell lines implicate NR5A1 as a direct transcriptional regulator of all three of these genes, as well as *Cga* [10], [11], [12], [13], [14], [15]. Nonetheless, *Nr5a1* knockout mice retain the ability to produce LH and FSH in response to exogenous GnRH stimulation [9], [16]. These data suggest that NR5A1 is dispensable for GnRH-stimulated gonadotropin production or that another (perhaps related) factor might substitute for its absence. Consistent with the latter possibility, mutation of a conserved “gonadotrope-specific element” (GSE or NR5A1 binding site) in the bovine *Lhb* promoter abolishes its GnRH responsiveness in transgenic mice [17]. Therefore, the available data collectively suggest a more important role for the GSE than for the NR5A1 protein itself in mediating the transcriptional response of the *Lhb* gene to GnRH.

NR5A2 (also known as liver receptor homolog 1; LRH-1) shares a high degree of sequence homology with NR5A1, binds the same consensus DNA sequence, and regulates many of the same genes [18], [19]. Despite their functional overlap, global and cell-specific knockout studies in mice clearly show that the two proteins play distinct roles [20], [21]. NR5A2 is expressed in adult murine pituitary gland and in immortalized gonadotrope-like cell lines, and can bind and activate the rat *Lhb* promoter *in vitro* [22], [23]. Here, we investigated NR5A2's regulation of basal and GnRH-stimulated murine *Lhb* and *Fshb* transcription in immortalized cells and then ablated the gene specifically in gonadotropes in mice. Whereas NR5A2 is a potent regulator of gonadotropin β subunit promoter activities *in vitro*, it is dispensable for normal gonadotrope function and fertility *in vivo*.

## Materials and Methods

### Reagents

DMEM with 4.5 g/l glucose, l-glutamine and sodium pyruvate was from Wisent (St-Bruno, Quebec, Canada). Lipofectamine, Plus reagent, Lipofectamine 2000, gentamycin, fetal bovine serum (FBS) and SYBR green quantitative PCR master mix were purchased from Invitrogen (Burlington, Ontario, Canada). Anti-FLAG antibody (F7425) and chemicals were from Sigma (St. Louis, MO). Taq polymerase, T4 DNA ligase, restriction endonucleases, deoxynucleotide triphosphates and 5× Passive Lysis Buffer (PLB) were from Promega (Madison, WI). Goat anti-rabbit IgG-HRP conjugate (170–6515) was from Bio-Rad (Hercules, CA). Protease inhibitor tablets (Complete-Mini) were from Roche (Indianapolis, IN). ECL-plus reagent and protein markers were from GE Healthcare (Piscataway, NJ). Oligonucleotides were synthesized by Integrated DNA Technologies (Coralville, IA). siRNAs for *Nr5a1* (D-051262-01; previously described and validated in [24]) and *Nr5a2* (siRNA #1: D-047044-03; siRNA#2: D-047044-04) were obtained from Dharmacon (Lafayette, CO).

### Cell culture, reporter assays, and western blot

LβT2 cells ([25]; gift from Dr. P. Mellon, University of California, San Diego) and CHO cells (ATCC CCL-61; gift from Dr. P. Morris, Population Council, New York, NY) were cultured as previously described [26], [27]. For reporter assays, LβT2 cells were seeded at 3×10^5^ cells per well in 48-well plates three days before transfection with Lipofectamine 2000. HeLa cells (ATCC CCL-2; gift from Dr J. Tanny, McGill University, Montréal) were cultured in DMEM supplemented with 10% FBS, and plated at 1.8×10^4^ cells per well in 48-well plate for reporter assays. Cells were transfected the next day using Lipofectamine 2000. Reporter assays were performed as previously described [28]. For western blots, CHO cells in 10-cm dishes were transfected using Lipofectamine following the manufacturer's instructions. Whole cell lysates were prepared and analyzed as described [26].

### Plasmids

The murine −232/+5 *Lhb*-luc and −1990/+1 *Fshb*-luc reporters, as well as the EGR1, NR5A1 and PITX1 expression vectors were described previously [24], [26], [29], [30]. The murine −1772/+38 *Gnrhr*-luc reporter was a gift from Colin Clay (Colorado State University, Fort Collins, CO). The full-length and short isoform (variant #2) NR5A2 expression vectors were produced by PCR amplification from full-length NR5A2 in pCMX [31] and sub-cloned in the *EcoR*I and *Xba*I sites of pcDNA3.0. The same strategy was used for generation of FLAG-tagged NR5A2 constructs, except that the forward primers replaced the translation initiation codon (ATG) with CGA, and the products were cloned in-frame downstream of a FLAG tag in pcDNA3.0 [30]. Mutant reporters and expression vectors were produced by site-directed mutagenesis following the QuikChange protocol (Stratagene).

### 5′ rapid amplification of cDNA ends (RACE)

5′RACE was performed using the FirstChoice RLM-RACE kit (Ambion, Austin, TX), following the manufacturer's protocol. Briefly, following 5′ RACE adapter ligation to decapped total murine pituitary RNA, first-strand cDNA synthesis was performed using an *Nr5a2* gene-specific primer in exon 3. Two rounds of nested PCR were performed using forward primers in the 5′RACE adapter and reverse gene-specific primers in exon 3 of *Nr5a2*. PCR products were cloned in pGEM-T Easy (Promega, Madison, WI) and sequenced (GenomeQuébec, Montréal).

### Animals

*Nr5a2^fl/fl^*, *Gnrhr^GRIC/+^,* and *ROSA26^eYFP/+^* mice were described previously [32], [33], [34]. To generate gonadotrope-specific *Nr5a2* knockout and control animals, *Nr5a2^fl/fl^;Gnrhr^+/+^* mice were bred with *Nr5a2^+/+^*;*Gnrhr^GRIC/GRIC^* mice. Resulting *Nr5a2^fl/+^;Gnrhr^GRIC/+^* females were crossed to *Nr5a2^fl/fl^; Gnrhr^+/+^* males to generate littermates with the experimental (*Nr5a2^fl/fl^*;*Gnrhr^GRIC/+^*) and control (*Nr5a2^fl/fl^*; *Gnrhr^+/+^*) genotypes. *Gnrhr^GRIC/+;^ROSA26^eYFP/+^* mice were generated by crossing *Gnrhr^GRIC/GRIC^;ROSA26^+/+^* females with *Gnrhr^+/+^;ROSA26^eYFP/eYFP^* males. *Nr5a2^fl/fl^;Gnrhr^GRIC/+^;ROSA26^eYFP/+^* were generated by crossing *Nr5a2^fl/fl^;Gnrhr^GRIC/+^;ROSA26^+/+^* females with *Nr5a2^fl/fl^;Gnrhr^+/+^;ROSA26^eYFP/eYFP^* males. Genotyping primers are listed in Table S1. For mating studies, 8 week-old experimental and control male or female mice were individually paired with a single adult C57BL6 mouse of the opposite sex, and fertility was evaluated over a period of six months. The presence of newborn mice was monitored daily starting from 20 days after pairing. Pups were counted immediately after birth. For tissue and blood collection, six-week old animals were used. All animal experiments were performed in accordance with institutional and federal guidelines and approved by the McGill University IACUC and the Comité d'Éthique de l'Utilisation des Animaux of the Université de Montréal.

### FACS sorting of primary gonadotrope cells

For gonadotrope purification, adult (>6 week-old) male and female *Nr5a2^fl/fl^;Gnrhr^GRIC/+^;ROSA26^eYFP/+^* and *Gnrhr^GRIC/+^;ROSA26^eYFP/+^* mice were sacrificed by CO_2_ asphyxiation. Dissected pituitaries were collected in M199 media containing 10% FBS, washed three times in HBSS, and minced with a scalpel in a Petri dish. Minced pituitaries were digested in 1.5 mg/mL collagenase (Sigma #C-0130; diluted in Hank's Balanced Salt Solution HBSS with 30 mg/mL BSA, pH 7.4) at 37°C for 2 h with gentle stirring (40 μL/pituitary). The tissue suspension was then washed with 10 mL calcium-free HBSS, centrifuged for 5 min at 1200× g, and resuspended in pancreatin solution (Sigma P3292; 4.5 mg/mL in calcium-free HBSS; 40 μL/pituitary). Pancreatin digestion was performed in a 37°C water bath with manual agitation for 15 min. The resulting cell suspension was washed three times in 10 mL M-199 media supplemented with 10% FBS, with centrifugation steps between each wash as above, and filtered with a 40 micron nylon mesh. The final cell pellet was resuspended in 1 mL M-199 media, and the cells sorted using a FACSAria cell sorter. Both YFP-positive (i.e., gonadotropes) and YFP-negative (i.e., non-gonadotropes) were used in subsequent analyses.

### RNA extraction, cDNA synthesis and qPCR

Total RNA was extracted from cells (Allprep DNA/RNA, Qiagen) or tissues (TRIzol, Invitrogen), following the manufacturer's instructions. cDNA was prepared as previously described [26]. Quantitative PCR (qPCR) was performed using the Platinum SYBR Green qPCR SuperMix-UDG (Invitrogen) on a Corbett Rotor-Gene 6000 instrument. Samples were assayed in duplicate or triplicate, and analyzed using the 2^−ΔΔCt^ method [35]. qPCR primers are listed in Table S1.

### Hormone assays

For serum collection, mice were killed by CO_2_ asphyxiation, and blood obtained by cardiac puncture. The blood was left to clot for 15 minutes at room temperature, and then spun at 3000 rpm for 10 minutes for serum isolation. Serum LH and FSH were measured using the mouse/rat LH/FSH multiplex assay at the Ligand Assay and Analysis Core of the University of Virginia Center for Research in Reproduction.

### Statistical analysis

Data from the cell culture experiments were analyzed using one- or two-way ANOVA, with Tukey post-hoc test to assess differences between groups. For all the reporter experiments, “N” is equal to the number of experiments. In some reporter experiments, data were log-transformed when variances were unequal between groups. Data from the animal experiments were analyzed using independent t-tests. *P*-values <0.05 were considered statistically significant.

## Results

### A short Nr5a2 mRNA isoform is expressed in the murine pituitary gland

**Figure 1:**
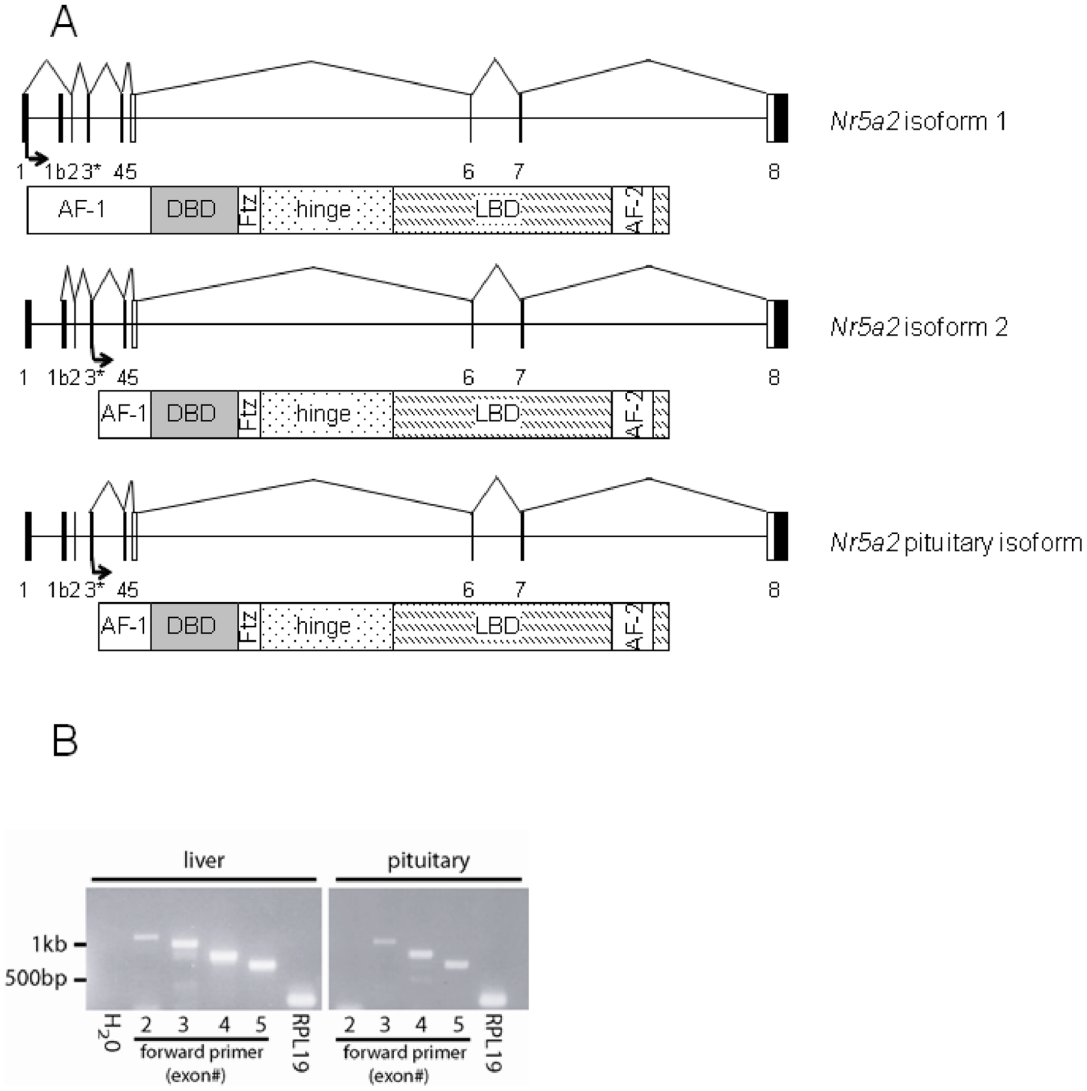
Characterization of a pituitary-specific *Nr5a2* mRNA isoform. **A**) Schematic representation of the *Nr5a2* locus, *Nr5a2* mRNA isoforms, and the resulting protein products. Black boxes indicate coding exons; white boxes indicate non-coding sequences. The asterisk (*) after exon 3 denotes the fact that it comes in two forms: the shorter, canonical form (denoted in black) first described in isoforms 1 and 2, and a 5′-extended form (denoted by the gray extension at the 5′ end) expressed in the pituitary (as mapped by 5′RACE in this study). Arrows indicate the alternative translation start sites in exons 1 and 3. AF-1, activation function 1; DBD, DNA-binding domain; Ftz, *fushi tarazu* F1-like box; LBD, ligand-binding domain; AF-2, activation function 2. **B**) PCR analysis of cDNA prepared from murine liver and pituitary. A common antisense primer in *Nr5a2* exon 6 was used in all the reactions. Sense primers were located in exon 2, 3, 4 or 5 as indicated. Amplification of *Rpl19* was used as a positive control for cDNA integrity.

Because previous results indicated that NR5A2 from whole pituitary or gonadotrope-like cell lines migrated faster than *in vitro* translated full-length (liver-derived) NR5A2 in western blots [22], we first mapped the transcription start site of *Nr5a2* in murine pituitary by 5′ rapid amplification of cDNA ends (5′ RACE). Analyses of several independent clones indicated that transcription is initiated in the second intron, 217 base pairs (bp) upstream of the third exon (Fig. 1A; GenBank acc. # JX648197). The resulting mRNA likely utilizes the previously described start of translation in exon 3 [36], producing an isoform lacking the N-terminal 61 amino acids of the full-length protein, which constitutes most of the AF1 domain (“NR5A2 variant #2”, CCDS acc. # NP_001153241). To confirm the 5′ RACE results, murine liver and pituitary cDNAs were analyzed by PCR using a reverse primer in exon 6 and forward primers in exons 2, 3, 4, or 5. Whereas a product was detected with all primer pairs using liver cDNA, no product was obtained from pituitary cDNA with the forward primer in exon 2, confirming that pituitary transcripts lack this exon (Fig. 1B). In subsequent experiments, we used an expression vector encoding the pituitary NR5A2 isoform.

### NR5A2 activates the murine Lhb promoter

**Figure 2:**
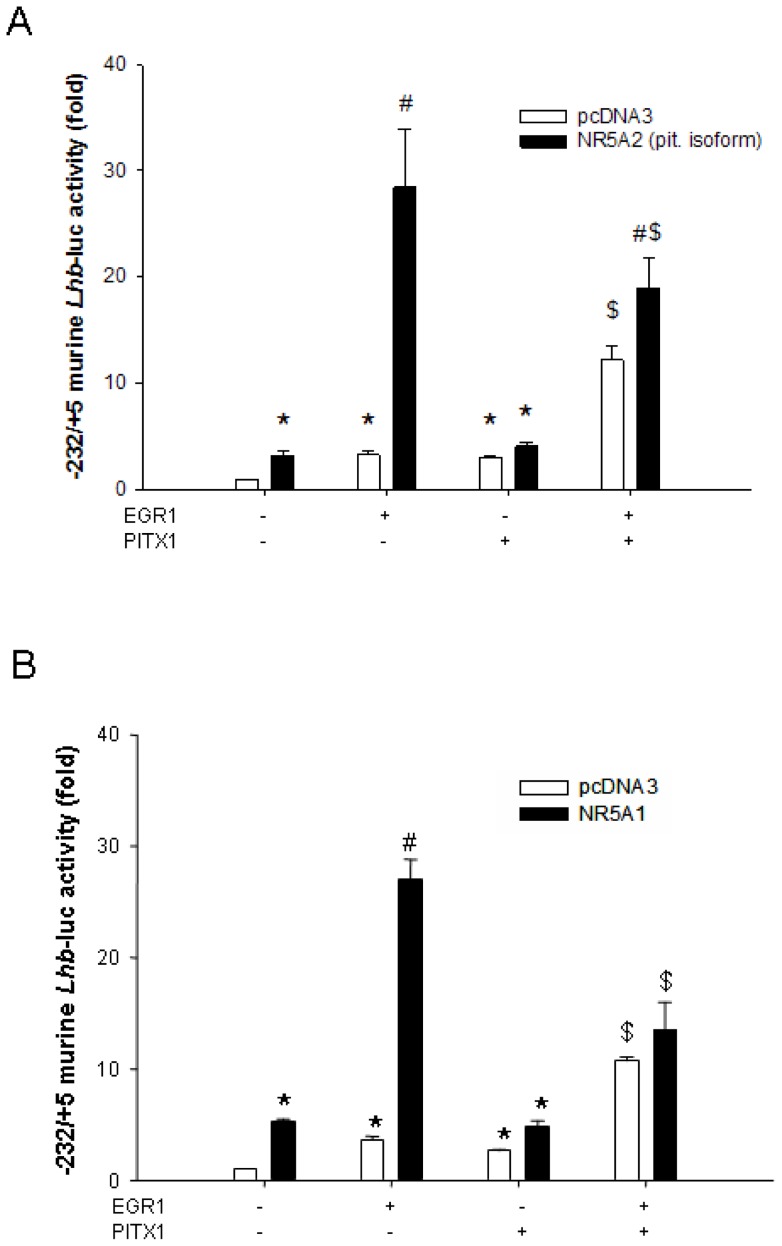
Activation of the murine *Lhb* promoter by NR5A2. HeLa cells were transfected with 225 ng/well of the murine −232/+5 *Lhb*-luc reporter as well as 50 ng/well of EGR1 and/or PITX1 expression constructs and 50 ng/well of **A**) NR5A2 (pituitary isoform) or **B**) NR5A1 expression vectors (black bars) or empty vector (pcDNA3 – white bars). Bars with different symbols differ significantly. Data represent the mean + SEM of four (**A**) or five (**B**) independent experiments performed in triplicate.

To determine whether NR5A2 can induce murine *Lhb* transcription, we examined the effect of ectopic NR5A2 expression on transcriptional activity of the proximal murine *Lhb* promoter in heterologous HeLa cells. NR5A2 robustly induced the murine -232/+5 *Lhb*-luc reporter (Fig. 2A) and did so to a similar extent as NR5A1 (Fig. 2B). NR5A1 cooperatively activates the *LHB*/*Lhb* promoter with early-growth response 1 (EGR1) and *paired*-like homeodomain (PITX) transcription factors [10], [24], [37], [38], [39], [40]. To assess whether NR5A2 can mediate similar functional interactions, we examined the effect of ectopically expressed NR5A1 or NR5A2 in combination with EGR1 and/or PITX1 on transcriptional activity of the murine *Lhb*-luc reporter in HeLa cells. All four factors individually activated the *Lhb* promoter, and EGR1 synergized with PITX1 (Fig. 2A–B). Furthermore, both NR5A1 and NR5A2: 1) synergized with EGR1, 2) had no significant effect on PITX1-mediated transcription, and 3) did not further amplify the synergism between EGR1 and PITX1 (Fig. 2A–B). Therefore, NR5A1 and NR5A2 produced indistinguishable functional interactions with EGR1 and PITX1 on the murine *Lhb* promoter in heterologous cells.

**Figure 3:**
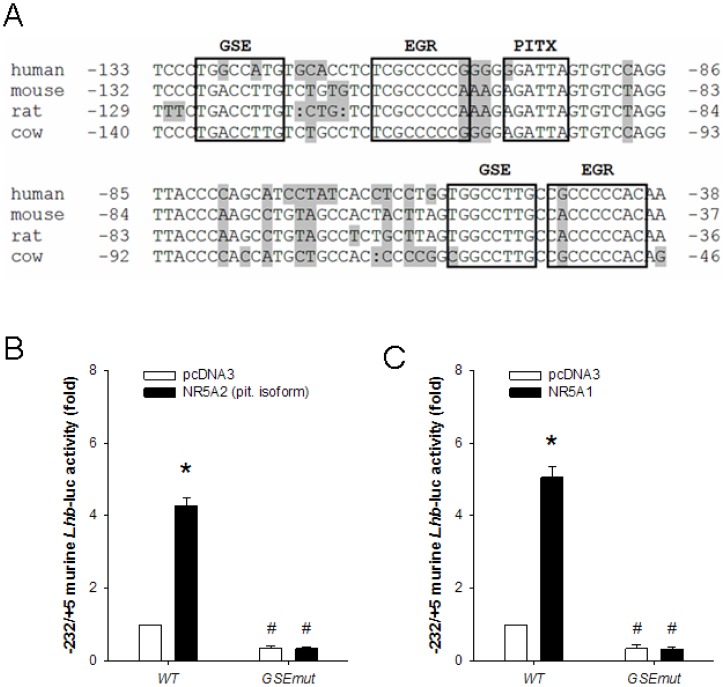
NR5A2 activate the murine *Lhb* promoter through conserved *GSE* elements. **A**) Alignment of the proximal *LHB*/*Lhb* promoters from human, mouse, rat and cow. Bases are numbered relative to the transcriptional start site (+1; not shown). The conserved GSE, *EGR* and *PITX* response elements are boxed. Nucleotides that differ between the species are shaded. **B**) and **C**) HeLa cells were transfected with 225 ng/well of the murine −232/+5 *Lhb*-luc reporter or the same reporter carrying a mutation in both GSE elements (*GSEmut*), along with 50 ng/well of **B**) NR5A2 (pituitary isoform) or **C**) NR5A1 expression vectors (black bars) or empty vector (pcDNA3 – white bars). Bars with different symbols differ significantly. Data represent the mean + SEM of five (**B**) and four (**C**) independent experiments performed in triplicate.

Activation of the *LHB*/*Lhb* promoter by NR5A1 requires two conserved response elements (GSEs at −128/−121 and −66/−59 relative to the transcription start site in mouse), which neighbor adjacent EGR1 and PITX binding sites in the proximal promoter ([10], [17], [24], [37], [38], [39], [40]; Fig. 3A). Because NR5A1 and NR5A2 share identical consensus DNA binding sites, we examined whether the NR5A2 response was also mediated through these two GSEs. To this end, we assessed the effect of ectopic NR5A2 expression on murine −232/+5 *Lhb* promoter-reporters lacking the distal, proximal, or both GSEs. Mutation of either one or both elements abolished induction of the promoter by NR5A1 or NR5A2 (Fig. 3B–C and Fig. S1).

Two putative GSEs (−341/−333 and −239/−231) in the murine *Fshb* promoter were previously shown to regulate basal promoter activity in cooperation with NF-Y binding sites [13]. However, direct actions of NR5A1 on *Fshb* transcription via these (or other) *cis*-elements were not demonstrated. Therefore, we examined the ability of NR5A1 or NR5A2 to induce the murine −1990/+1 *Fshb*-luc reporter in HeLa cells. Unlike the case with the *Lhb* reporter, neither NR5A1 nor NR5A2 activated *Fshb* promoter activity (Fig. S2). Co-expression of NR5A1 or NR5A2 with PITX1 also failed to stimulate the *Fshb* promoter (data not shown).

### Endogenous NR5A2 regulates basal and GnRH-induced Lhb and Fshb promoter activities in LβT2 cells

**Figure 4:**
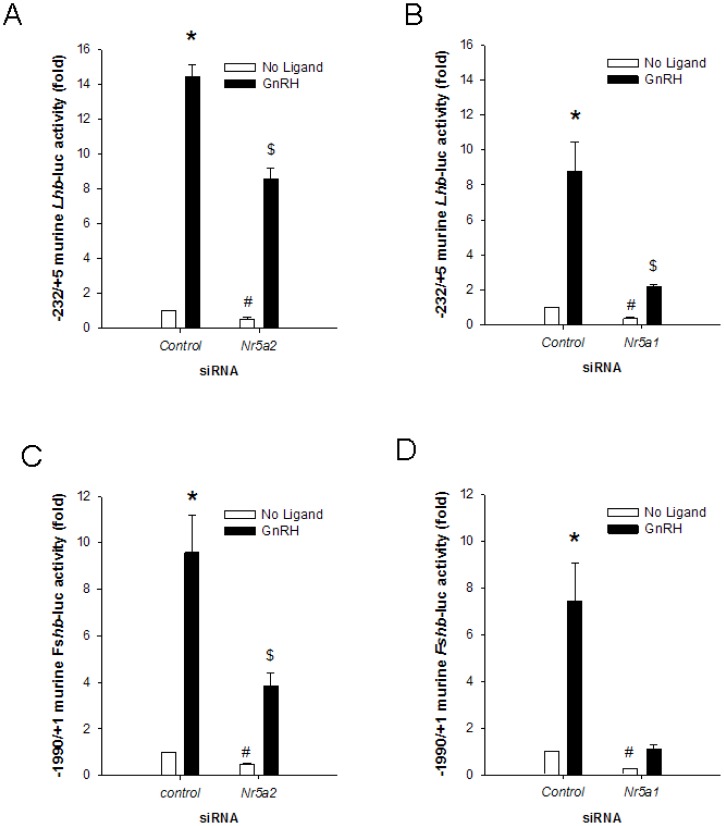
Endogenous NR5A2 regulates *Lhb* and *Fshb* promoter activity in immortalized gonadotropes. LβT2 cells were transfected with 225 ng/well of the following reporters: **A**) and **B**) murine −232/+5 *Lhb*-luc; **C**) and **D**) murine −1990/+1 *Fshb*-luc. Cells were co-transfected with the following siRNAs, at a final concentration of 5×10^−9^ M: **A**) and **C**) *Nr5a2*; **B**) and **D**) *Nr5a1*. In all cases, a non-specific siRNA, added at a final concentration of 5×10^−9^ M, was used as control. Cells were treated with 10^−7^ M GnRH for 6 h (black bars) or left untreated (white bars) prior to collection of whole cell lysates for luciferase assays. Bars with different symbols differ significantly. Data represent the mean +SEM of three (**A**, **B**, **D**), or seven (**C**) independent experiments performed in triplicate.

Next, we evaluated the role of endogenous NR5A1 or NR5A2 in basal and GnRH-stimulated transcriptional activity of the murine *Lhb* and *Fshb* promoters in homologous LβT2 cells. We co-transfected cells with the murine −232/+5 *Lhb*-luc reporter and short interfering RNAs (siRNAs) directed at *Nr5a1*, *Nr5a2* (isoform #2), or with a control siRNA, and stimulated with GnRH. *Nr5a1* and *Nr5a2* siRNAs significantly decreased basal and GnRH-stimulated promoter activity (Fig. 4A–B). The efficiency and sequence specificity of the siRNAs was verified in control experiments (Fig. S3A and Ref. [24]). In addition, one *Nr5a2* siRNA, which displayed poor efficiency at knocking down NR5A2 (siRNA #2), did not affect GnRH-stimulated promoter activity (Fig. S3B–C).

Although ectopically expressed NR5A1 or NR5A2 failed to activate *Fshb* promoter activity in heterologous HeLa cells (Fig. S2), we examined the effect of depleting endogenous NR5A1 or NR5A2 on the activity of the murine −1990/+1 *Fshb*-luc reporter in LβT2 cells. Similar to the *Lhb* promoter, both the *Nr5a1* and *Nr5a2* siRNAs significantly decreased basal and GnRH-stimulated activation of the −1990/+1 *Fshb* promoter (Fig. 4C–D). As observed with the *Lhb* promoter, *Nr5a2* siRNA #2 did not significantly impair GnRH stimulation of the *Fshb* promoter (Fig. S3D).

### Generation of gonadotrope-specific Nr5a2 knockout mice

**Figure 5:**
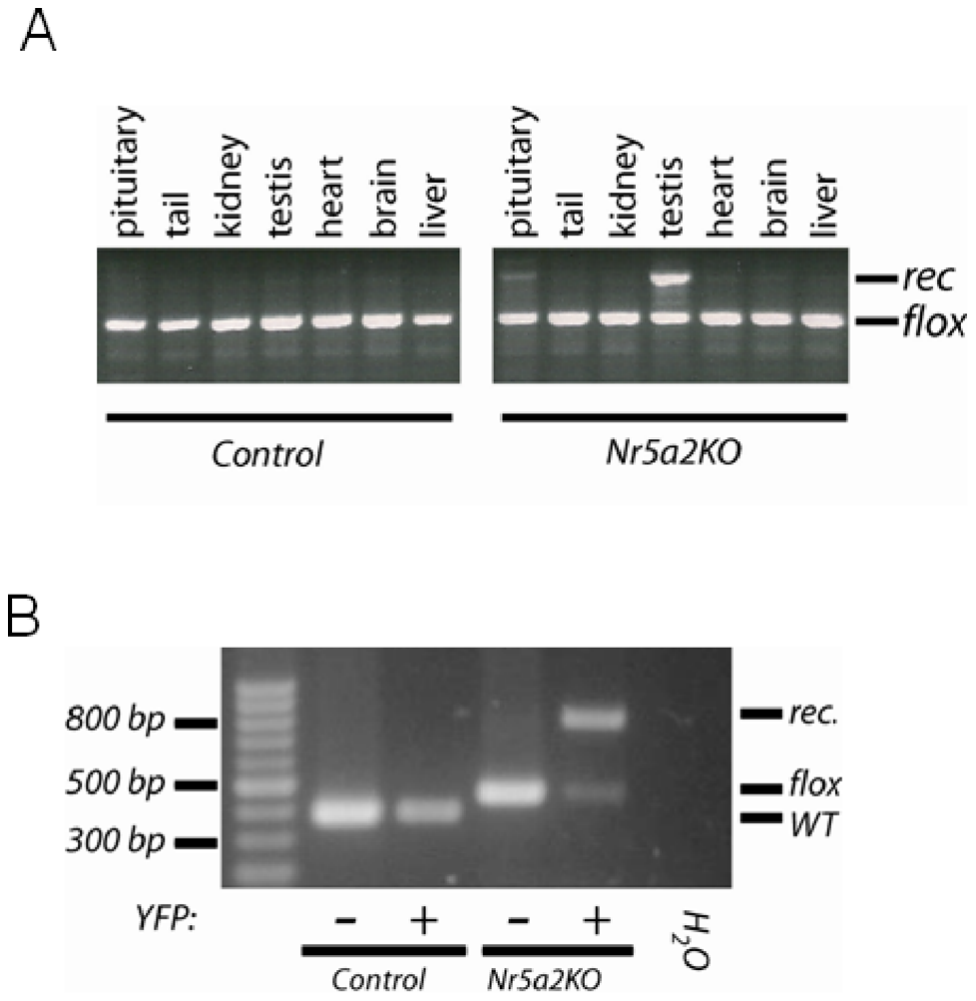
Generation and validation of gonadotrope-specific *Nr5a2* knockout mice. **A**) Genomic DNA was extracted from the indicated tissues of *Nr5a2^fl/fl^* (control) and *Nr5a2^fl/fl^*; *Gnrhr^GRIC/+^* (*Nr5a2KO*) mice and analyzed by PCR for the presence of the floxed (“flox” – lower band) or recombined (“rec” – upper band) *Nr5a2* alleles. **B**) Pituitary cells from *Gnrhr^GRIC/+^*;*ROSA26^eYFP/+^* (control) and *Nr5a2^fl/fl^*;*Gnrhr^GRIC/+^*;*ROSA26^eYFP/+^* (*Nr5a2*KO) mice were sorted by FACS, and genomic DNA was extracted from the YFP-positive (+) and YFP-negative (−) fractions. Genotyping PCR was performed to detect the presence of the wild-type (“WT”), “floxed” (“flox”) and “recombined” (“rec”) *Nr5a2* alleles.

The *in vitro* data above show that NR5A2 is expressed in pituitary and functions similarly to NR5A1 with respect to *Lhb* promoter activity. To test whether NR5A2, like NR5A1, plays an essential role in gonadotropin synthesis and fertility *in vivo*, we generated mice with a gonadotrope-specific deletion of *Nr5a2* by crossing *Gnrhr^GRIC^* mice with *Nr5a2^fl/fl^* mice. As loxP sites flank exons 4 and 5 in this *Nr5a2* allele, these mice are expected to lack both the full-length and shorter NR5A2 isoforms. Recombination of the *Nr5a2* gene was only observed in pituitary gland and in testes (Fig. 5A), as expected from the previously described expression pattern of Cre recombinase in *Gnrhr^GRIC^* mice [41]. To assess the efficiency of recombination of *Nr5a2* in our model, we bred the *ROSA26^eYFP^* reporter allele [33] into the *Nr5a2^fl/fl^*;*Gnrhr^GRIC/+^* background. In the resulting mice, expression of Cre recombinase in gonadotropes results in the simultaneous deletion of the *Nr5a2* gene and expression of YFP. We purified gonadotropes (YFP + cells) from *Nr5a2^fl/fl^*;*Gnrhr^GRIC/+^*;*ROSA26^eYFP/+^* mice by FACS (Fig. S4) and examined the extent of *Nr5a2* recombination by PCR. YFP + cells showed the expected PCR product for the recombined allele, indicating the near complete deletion of *Nr5a2* in those cells (Fig. 5B). By comparison, cells in the YFP- fraction only showed a band corresponding to the “floxed” (non-recombined) *Nr5a2* allele. Therefore, *Nr5a2* was efficiently and specifically recombined in gonadotropes in our model.

### Normal gonadotropin synthesis and fertility in gonadotrope-specific Nr5a2 knockout mice

**Figure 6:**
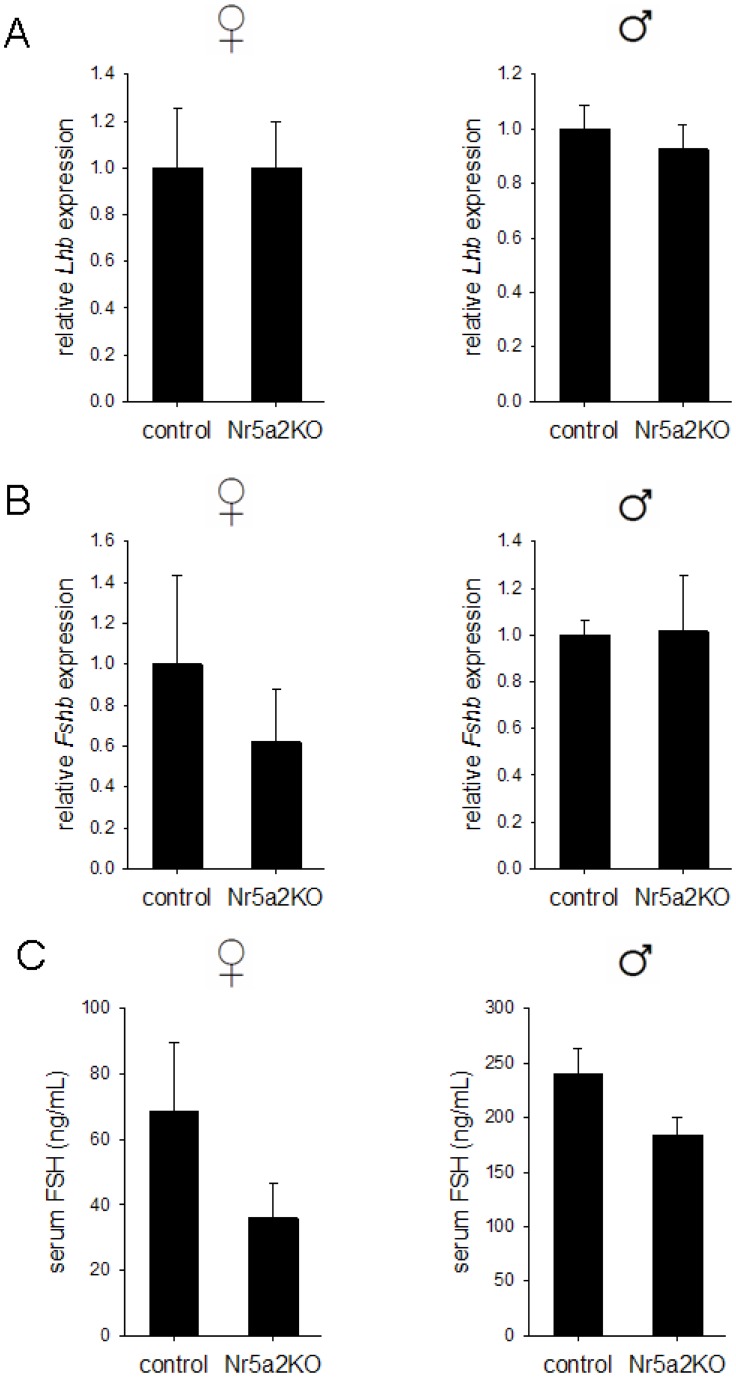
Normal gonadotropin synthesis in gonadotrope-specific *Nr5a2* knockout mice. cDNA was prepared from total RNA isolated from individual pituitary glands of *Nr5a2^fl/fl^* (control) and *Nr5a2^fl/fl^*;*Gnrhr^GRIC/+^* (*Nr5a2*KO) mice, and analyzed for expression of **A**) *Lhb* and **B**) *Fshb* by quantitative real-time PCR. The amount of *Lhb* and *Fshb* transcripts was normalized to the expression of the housekeeping gene *Rpl19*. For **A**) and **B**), n = 6 for control and n = 7 for *Nr5a2*KO mice, for both males and females. **C**) Serum FSH levels were measured in 6-week old male (control, n = 5; *Nr5a2*KO, n = 7) and female (control, n = 4; *Nr5a2*KO, n = 6) mice.

As our *in vitro* data suggested a role for NR5A2 in the regulation of both *Lhb* and *Fshb* transcription, we examined the expression of these genes in the pituitaries of 6 week-old *Nr5a2^fl/fl^*; *Gnrhr^GRIC/+^* (hereafter *Nr5a2*KO) mice and *Nr5a2^fl/fl^* (hereafter “control”) littermates. These analyses revealed no significant difference in the expression of either *Lhb* or *Fshb* between genotypes, both in males and females (Fig. 6A–B). Accordingly, serum levels of FSH were normal in *Nr5a2*KO male and female mice, whereas LH levels were highly variable and often undetectable in mice of both sexes and genotypes (Fig. 6C and data not shown). Next, we assessed reproductive function in *Nr5a2*KO mice by monitoring the fertility of males and females after pairing with wild-type C57BL/6 control mice over a period of 6 months. These studies revealed normal fertility in *Nr5a2*KO mice, as neither males nor females differed from controls in terms of mean litter size, inter-litter interval, latency to first litter, or cumulative number of pups produced over the duration of the mating trial (Table 1). Finally, we examined gonads and accessory sex organs in *Nr5a2*KO mice (testes and seminal vesicles in males; ovaries and uteri in females). All of the examined tissues were normal in appearance (data not shown) in *Nr5a2*KO mice and did not differ in weight relative to controls (Fig. S5). Collectively, these results indicate that the activity of the reproductive axis and the production of gonadotropins are intact in gonadotrope-specific *Nr5a2* knockout mice.

**Table 1:** Fertility data.

Genotype	N	Days to first litter	Mean litter size	Average number of pups	Inter-litter interval (days)
**males**					
*Control*	5	26.2±5.2	5.3±0.2	24.4±3.8	30.8±2.5
*Nr5a2*KO	5	31.2±4.2	6.1±0.6	33.0±3.6	27.0±2.2
**females**					
*Control*	5	26.2±1.3	7.3±0.4	40.8±3.2	29.2±0.7
*Nr5a2*KO	7	26.4±6.9	6.6±0.7	38.1±2.8	25.6±1.9

Mice of the indicated genotypes were individually paired with control (C57BL6) mice of the opposite sex for a period of 6 months, and fertility parameters were recorded. Data represent mean ± SEM.

### Nr5a2 is expressed at low levels in murine gonadotropes

**Figure 7:**
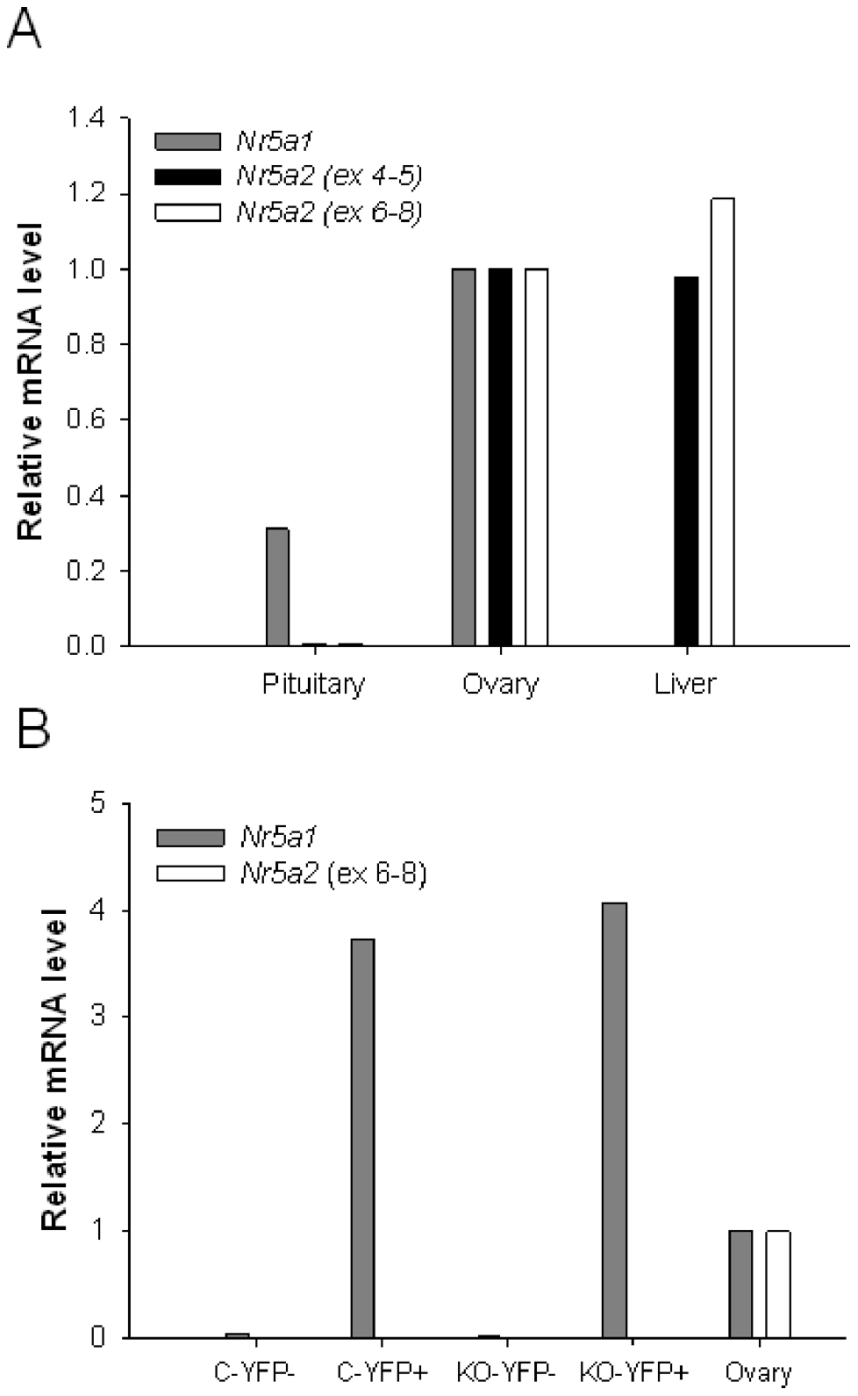
*Nr5a2* is expressed at low levels in gonadotropes. **A**) cDNA was prepared from total RNA isolated from the indicated tissues from wild-type mice, and analyzed for the expression of *Nr5a1* or *Nr5a2*. Two different primer sets were used for Nr5a2, with the first using a forward primer in exon 4 and a reverse primer in exon 5 (ex 4–5) and the second with a forward primer in exon 6 and a reverse primer in exon 8 (ex 6–8). **B**) cDNA was prepared from total RNA isolated from sorted cells from *Gnrhr^GRIC/+^*;*ROSA26^eYFP/+^* mice (C-YFP-: YFP-negative cells ; C-YFP +: YFP-positive cells; C = control), *Nr5a2^fl/fl^*;*Gnrhr^GRIC/+^*;*ROSA26^eYFP/+^* mice (KO-YFP-: YFP-negative cells; KO-YFP+: YFP-positive cells; KO = knockout), or wild-type mouse ovary. Expression of *Nr5a1* and *Nr5a2* (exon 6–8 primer set) was assessed by qPCR, and normalized to the expression of the housekeeping gene *Rpl19*. In both panels, the results for ovary were normalized to 1 for each primer set and the values for other tissues or isolated cells presented relative to ovary.

Though our 5′ RACE results demonstrated *Nr5a2* mRNA expression in whole pituitary, the absence of a reproductive phenotype in gonadotrope-specific *Nr5a2* knockout mice led us to ask whether the gene is actually expressed in gonadotropes. We first attempted to localize the NR5A2 protein in adult pituitary by immunohistochemistry or immunofluorescence, but were unable to produce consistent results (data not shown). Therefore, we focused on mRNA expression. We developed qPCR assays using previously validated primers [32] as well as primers within the exons 4 and 5, which are deleted upon recombination in *Nr5a2* floxed mice. *Nr5a2* is robustly expressed in liver and ovary and we confirmed these observations with both primer sets and total RNA extracted from these tissues in wild-type mice (Fig. 7A). In whole pituitary, *Nr5a2* was expressed at only 0.6% the level observed in ovary (Fig. 7A). Using the same cDNA samples, we readily detected *Nr5a1* in ovary and pituitary, but not liver (Fig. 7A). To more directly assess expression in gonadotropes, we purified cells from *Gnrhr^GRIC/+^;ROSA26^eYFP/+^* (control) and *Nr5a2^fl/fl^;Gnrhr^GRIC/+^;ROSA26^eYFP/+^* (*Nr5a2* KO). Whereas *Nr5a1* mRNA was enriched in YFP + (i.e., gonadotropes) relative to YFP- (i.e., non-gonadotropes) cells, *Nr5a2* was uniformly low in both cell populations in both the control and knockout genotypes (Fig. 7B). In fact, the levels were so low as to preclude a reliable assessment of the extent of mRNA depletion in knockouts. That said, our analysis of DNA from the same purified cells clearly shows how efficiently the floxed allele was recombined (Fig. 5B). Collectively, these data suggest that *Nr5a2* is expressed at very low levels in gonadotropes *in vivo*.

## Discussion

Here, we characterized the role of NR5A2 in gonadotropin subunit expression *in vitro* and *in vivo*. We identified a novel, pituitary-specific *Nr5a2* mRNA isoform in murine pituitary gland. The encoded NR5A2 protein lacks the first 61 N-terminal amino acids of the full-length (canonical) protein, thus truncating most of its “activator function 1” (AF-1) domain. In contrast to other nuclear receptors, no specific function has been ascribed to this domain in NR5A2, and all NR5A2 co-regulators characterized to date interact via its C-terminal AF-2 region [18], [42]. Accordingly, we observe that the full-length and pituitary NR5A2 isoforms are functionally indistinguishable in their abilities to activate the murine *Lhb* promoter (data not shown) and do so to an extent and in a manner comparable to NR5A1. Indeed, the NR5A2 response is mediated through the same proximal promoter *cis*-elements (GSEs) used by NR5A1 [10], [24], [37], [38], [39], [40]. Furthermore, NR5A2 functionally interacts with other well-characterized transcriptional regulators of *Lhb*, EGR1 and PITX1 [2], [24], [40], [43], in an identical manner to NR5A1. By knockdown experiments in the LβT2 murine gonadotrope-like cell line, we also show that endogenous NR5A2 regulates both basal and GnRH-stimulated *Lhb* promoter activation. However, the effect of NR5A1 depletion is more dramatic than that of NR5A2. At least part of the difference might be attributable to the relative roles of NR5A1 and NR5A2 in GnRH receptor expression. Whereas *Nr5a1* knockdown significantly impairs *Gnrhr* promoter-reporter activity, *Nr5a2* siRNAs has little to no effect (Fig. S6A). Similarly, whereas *Gnrhr* mRNA levels are significantly depleted in pituitaries of *Nr5a1* knockout mice [9], *Gnrhr* expression is normal in our *Nr5a2*KO animals (Fig. S6B). These data suggest that NR5A1 and NR5A2 play similar, though not identical roles in gonadotrope-like cells.

The data also show that depletion of either NR5A1 or NR5A2 in LβT2 cells substantially impairs basal and GnRH-stimulated murine *Fshb* promoter-reporter activity. Exactly how NR5A1 and/or NR5A2 regulate *Fshb* expression is presently unclear. Although previous work suggested a requirement for putative NR5A1 binding sites within the proximal promoter for basal *Fshb* reporter activity [13], a role for the NR5A1 protein itself was not demonstrated in that study. Further, it was previously reported that NR5A1 does not activate the bovine *Fshb* promoter by itself [44]. In our hands, neither NR5A1 nor NR5A2 induce murine *Fshb* promoter-reporter activity when ectopically expressed in heterologous reporter assays. However, the dramatic loss of *Fshb* mRNA and FSH protein in gonadotropes of pituitary-specific *Nr5a1* knockout mice [9] certainly suggests a role, either direct or indirect, for NR5A1 in *Fshb* expression.

Our findings in heterologous and gonadotrope-like cell lines prompted us to investigate the role of NR5A2 in gonadotrope function *in vivo*. To this end, we generated and analyzed gonadotrope-specific *Nr5a2* knockout (*Nr5a2*KO) mice. We confirm the efficient recombination of the *Nr5a2* gene in gonadotropes, in good agreement with the previously demonstrated efficiency and specificity of the *Gnrhr^GRIC^* allele we used to target Cre expression [34], [41], [45] Nonetheless, *Nr5a2*KO mice show normal *Lhb* and *Fshb* mRNA expression and circulating gonadotropin levels, and are fertile. Though expressed and functional in LβT2 cells, *Nr5a2* mRNA levels are extremely low in actual adult gonadotropes. Thus, the most likely explanation for our results is that, among NR5A family members, NR5A1 (which is maintained at normal levels in our mice), but not NR5A2 is the major regulator of gonadotropin synthesis *in vivo*.

In summary, we provide *in vitro* evidence that NR5A2 can mediate transcriptional activation of the murine *Lhb* and *Fshb* promoters by GnRH. Moreover, NR5A2 appears to regulate *Lhb* transcription in a manner analogous to NR5A1. However, unlike the case in *Nr5a1*-deficient mice, gonadotrope-specific *Nr5a2*KO animals exhibit normal gonadotropin subunit expression and reproductive function. These observations underscore the importance of *in vivo* validation of models developed exclusively in immortalized cell lines.

## Supporting Information

Figure S1 **Both conserved GSE elements mediate NR5A2 activation of the murine** ***Lhb*** **promoter.** HeLa cells were transfected with 225 ng/well of the murine −232/+5 *Lhb*-luc reporter or the same reporter carrying a mutation in the proximal (*pGSEmut*) or distal (*dGSEmut*) GSE elements along with 50 ng/well of **A**) NR5A2 (pituitary isoform) or **B**) NR5A1 expression vectors (black bars) or empty vector (pcDNA3 – white bars). Bars with different symbols differ significantly. Data represent the mean +SEM of five (**A**) and four (**B**) independent experiments performed in triplicate. Note that these data are from the same experiments as those shown in Figure 3. Therefore, the values for the wild-type (WT) reporter are the same in both figures.
(TIF)

Figure S2 **NR5A1 and NR5A2 do not directly activate the murine** ***Fshb*** **promoter.** HeLa cells were transfected with 225 ng/well of the murine −232/+5 *Lhb*-luc or −1990/+1 *Fshb*-luc reporter as well as 50 ng/well of **A**) NR5A2 or **B**) NR5A1 (pituitary isoform) expression vectors or empty vector (pcDNA3). Bars with different symbols differ significantly. Data represent the mean + SEM of three independent experiments performed in triplicate.
(TIF)

Figure S3 **Validation of the** ***Nr5a2*** **siRNAs used in this study. A**) and **B**) CHO cells were transfected with wild-type (WT) or siRNA-resistant (Res.) forms of Flag-tagged NR5A2 along with 5×10−9 M non-specific (control) or *Nr5a2* siRNAs, or 1X siRNA dilution buffer. Whole-cell lysates were collected and subjected to anti-Flag (top panel) or anti-β-actin (bottom panel) western blot analyses. **C**) and **D**) LβT2 cells were transfected with 225 ng/well **C**) murine −232/+5 *Lhb*-luc or **D**) murine −1990/+1 *Fshb*-luc reporters. Cells were co-transfected with *Nr5a2* siRNA #2, or a non-specific siRNA (control) at a final concentration of 5×10−9 M. Cells were treated with 10−7 M GnRH for 6 h (black bars) or left untreated (white bars) prior to collection of whole cell lysates for luciferase assays. Bars with different symbols differ significantly. Data represent the mean +SEM of three (**C**), or seven (**D**) independent experiments performed in triplicate. The data presented in panels **C** and **D** of this figure are from the same experiments as those of Figure **4A and 4C**, respectively. Therefore, the values for the “control” siRNA condition in the two figures are the same.
(TIF)

Figure S4 **Efficient and selective purification of YFP + gonadotropes from** ***Gnrhr^GRIC^*** ^**/+**^ **;** ***ROSA26^eYFP^*** ^**/+**^ **mice.** Dissociated pituitary cells from wild-type (**A,** ***a***) and *Gnrhr^GRIC^*/+;*ROSA26^eYFP^*/+ mice (**B, b–D, d**) were plated in primary culture. **A–D**) Pictures of cells taken under brightfield illumination. **a–d**) The same cells as in **A–D**, but viewed under fluorescent illumination for detection of YFP. **B, b**) Cells from GnrhrGRIC/+; *ROSA26^eYFP^*/+ mice prior to FACS. **C, c**) Cells from the YFP + fraction following FACS. **D, d**) Cells from the YFP- fraction following FACS. A field with a single YFP + cell is shown, but most fields examined lacked YFP + cells.
(TIF)

Figure S5 **Reproductive organ weights in gonadotrope-specific** ***Nr5a2*** **knockout mice.** Ovarian (**A**) and uterine (**B**) weights were measured in 6 week-old female *Nr5a2^fl/fl^* (control, n = 6) and *Nr5a2^fl/fl^*;*Gnrhr^GRIC^*/+ (*Nr5a2*KO, n = 6) mice. Testicular (**C**) and seminal vesicle (**D**) weights were measured in 6 week-old male mice (control, n = 8; *Nr5a2*KO, n = 8, bars  =  means).
(TIF)

Figure S6 ***Nr5a2*** **does not regulate gonadotrope expression of** ***Gnrhr in vitro*** **or** ***in vivo*** **. A**) LβT2 cells were transfected with 225 ng/well of the −1772/+38 murine *Gnrhr*-luc reporter. Cells were co-transfected with control, *Nr5a1* or *Nr5a2* siRNAs as indicated, at a final concentration of 5×10−9 M. Data represent the mean + SEM of four independent experiments performed in triplicate. **B**) cDNA was prepared from total RNA isolated from individual pituitary glands of *Nr5a2^fl/fl^* (control, n = 5) and *Nr5a2^fl/fl^*;*Gnrhr^GRIC^*/+ (*Nr5a2*KO, n = 8) male mice, and analyzed for expression of *Gnrhr* by quantitative real-time PCR.
(TIF)

Table S1 **Primer sequences.**
(TIF)

## References

[1] Bernard DJ, Fortin J, Wang Y, Lamba P (2010) Mechanisms of FSH synthesis: what we know, what we don't, and why you should care. Fertil Steril 93: 2465–2485. 20403589 10.1016/j.fertnstert.2010.03.034

[2] Jorgensen JS, Quirk CC, Nilson JH (2004) Multiple and Overlapping Combinatorial Codes Orchestrate Hormonal Responsiveness and Dictate Cell-Specific Expression of the Genes Encoding Luteinizing Hormone. Endocr Rev 25: 521–542. 15294880 10.1210/er.2003-0029

[3] Bliss SP, Navratil AM, Xie J, Roberson MS (2010) GnRH signaling, the gonadotrope and endocrine control of fertility. Front Neuroendocrinol 31: 322–340. 20451543 10.1016/j.yfrne.2010.04.002 PMC2923852

[4] Gharib SD, Wierman ME, Shupnik MA, Chin WW (1990) Molecular biology of the pituitary gonadotropins. Endocr Rev 11: 177–199. 2108012 10.1210/edrv-11-1-177

[5] Edson MA, Nagaraja AK, Matzuk MM (2009) The mammalian ovary from genesis to revelation. Endocr Rev 30: 624–712. 19776209 10.1210/er.2009-0012 PMC2761115

[6] Ma X, Dong Y, Matzuk MM, Kumar TR (2004) Targeted disruption of luteinizing hormone beta-subunit leads to hypogonadism, defects in gonadal steroidogenesis, and infertility. Proc Natl Acad Sci U S A 101: 17294–17299. 15569941 10.1073/pnas.0404743101 PMC535369

[7] Bliss SP, Miller A, Navratil AM, Xie J, McDonough SP, et al (2009) ERK signaling in the pituitary is required for female but not male fertility. Mol Endocrinol 23: 1092–1101. 19372235 10.1210/me.2009-0030 PMC2703601

[8] Liu F, Austin DA, Mellon PL, Olefsky JM, Webster NJ (2002) GnRH activates ERK1/2 leading to the induction of c-fos and LHbeta protein expression in LbetaT2 cells. Mol Endocrinol 16: 419–434. 11875099 10.1210/mend.16.3.0791

[9] Zhao L, Bakke M, Krimkevich Y, Cushman LJ, Parlow AF, et al (2001) Steroidogenic factor 1 (SF1) is essential for pituitary gonadotrope function. Development 128: 147–154. 11124111 10.1242/dev.128.2.147

[10] Halvorson LM, Kaiser UB, Chin WW (1996) Stimulation of luteinizing hormone beta gene promoter activity by the orphan nuclear receptor, steroidogenic factor-1. J Biol Chem 271: 6645–6650. 8636081 10.1074/jbc.271.12.6645

[11] Pincas H, Amoyel K, Counis R, Laverriere JN (2001) Proximal cis-acting elements, including steroidogenic factor 1, mediate the efficiency of a distal enhancer in the promoter of the rat gonadotropin-releasing hormone receptor gene. Mol Endocrinol 15: 319–337. 11158337 10.1210/mend.15.2.0593

[12] Fowkes RC, Desclozeaux M, Patel MV, Aylwin SJ, King P, et al (2003) Steroidogenic factor-1 and the gonadotrope-specific element enhance basal and pituitary adenylate cyclase-activating polypeptide-stimulated transcription of the human glycoprotein hormone alpha-subunit gene in gonadotropes. Mol Endocrinol 17: 2177–2188. 12920232 10.1210/me.2002-0393

[13] Jacobs SB, Coss D, McGillivray SM, Mellon PL (2003) Nuclear factor Y and steroidogenic factor 1 physically and functionally interact to contribute to cell-specific expression of the mouse Follicle-stimulating hormone-beta gene. Mol Endocrinol 17: 1470–1483. 12730328 10.1210/me.2002-0286 PMC2933173

[14] Barnhart KM, Mellon PL (1994) The orphan nuclear receptor, steroidogenic factor-1, regulates the glycoprotein hormone alpha-subunit gene in pituitary gonadotropes. Mol Endocrinol 8: 878–885. 7527122 10.1210/mend.8.7.7527122

[15] Duval DL, Nelson SE, Clay CM (1997) A binding site for steroidogenic factor-1 is part of a complex enhancer that mediates expression of the murine gonadotropin-releasing hormone receptor gene. Biol Reprod 56: 160–168. 9002645 10.1095/biolreprod56.1.160

[16] Ikeda Y, Luo X, Abbud R, Nilson JH, Parker KL (1995) The nuclear receptor steroidogenic factor 1 is essential for the formation of the ventromedial hypothalamic nucleus. Mol Endocrinol 9: 478–486. 7659091 10.1210/mend.9.4.7659091

[17] Keri RA, Nilson JH (1996) A steroidogenic factor-1 binding site is required for activity of the luteinizing hormone beta subunit promoter in gonadotropes of transgenic mice. J Biol Chem 271: 10782–10785. 8631889 10.1074/jbc.271.18.10782

[18] Fayard E, Auwerx J, Schoonjans K (2004) LRH-1: an orphan nuclear receptor involved in development, metabolism and steroidogenesis. Trends Cell Biol 14: 250–260. 15130581 10.1016/j.tcb.2004.03.008

[19] Yazawa T, Inaoka Y, Okada R, Mizutani T, Yamazaki Y, et al (2010) PPAR-gamma coactivator-1alpha regulates progesterone production in ovarian granulosa cells with SF-1 and LRH-1. Mol Endocrinol 24: 485–496. 20133449 10.1210/me.2009-0352 PMC5419099

[20] Duggavathi R, Volle DH, Mataki C, Antal MC, Messaddeq N, et al (2008) Liver receptor homolog 1 is essential for ovulation. Genes Dev 22: 1871–1876. 18628394 10.1101/gad.472008 PMC2492734

[21] Pelusi C, Ikeda Y, Zubair M, Parker KL (2008) Impaired follicle development and infertility in female mice lacking steroidogenic factor 1 in ovarian granulosa cells. Biol Reprod 79: 1074–1083. 18703422 10.1095/biolreprod.108.069435 PMC2780474

[22] Zheng W, Yang J, Jiang Q, He Z, Halvorson LM (2007) Liver receptor homologue-1 regulates gonadotrope function. J Mol Endocrinol 38: 207–219. 17293441 10.1677/jme.1.00001

[23] Lo A, Zheng W, Gong Y, Crochet JR, Halvorson LM (2011) GATA transcription factors regulate LHbeta gene expression. J Mol Endocrinol 47: 45–58. 21571865 10.1530/JME-10-0137

[24] Fortin J, Lamba P, Wang Y, Bernard DJ (2009) Conservation of mechanisms mediating gonadotrophin-releasing hormone 1 stimulation of human luteinizing hormone beta subunit transcription. Mol Hum Reprod 15: 77–87. 19106114 10.1093/molehr/gan079 PMC2734162

[25] Alarid ET, Windle JJ, Whyte DB, Mellon PL (1996) Immortalization of pituitary cells at discrete stages of development by directed oncogenesis in transgenic mice. Development 122: 3319–3329. 8898243 10.1242/dev.122.10.3319

[26] Bernard DJ (2004) Both SMAD2 and SMAD3 mediate activin-stimulated expression of the follicle-stimulating hormone beta subunit in mouse gonadotrope cells. Mol Endocrinol 18: 606–623. 14701940 10.1210/me.2003-0264

[27] Lamba P, Santos MM, Philips DP, Bernard DJ (2006) Acute regulation of murine follicle-stimulating hormone beta subunit transcription by activin A. J Mol Endocrinol. 36: 201–220. 10.1677/jme.1.01961 16461939

[28] Wang Y, Fortin J, Lamba P, Bonomi M, Persani L, et al (2008) Activator protein-1 and smad proteins synergistically regulate human follicle-stimulating hormone beta-promoter activity. Endocrinology 149: 5577–5591. 18653705 10.1210/en.2008-0220 PMC2584589

[29] Fortin J, Bernard DJ (2010) SMAD3 and EGR1 physically and functionally interact in promoter-specific fashion. Cell Signal 22: 936–943. 20149866 10.1016/j.cellsig.2010.01.019

[30] Lamba P, Khivansara V, D'Alessio AC, Santos MM, Bernard DJ (2008) Paired-like homeodomain transcription factors 1 and 2 regulate follicle-stimulating hormone beta-subunit transcription through a conserved cis-element. Endocrinology 149: 3095–3108. 18339718 10.1210/en.2007-0425 PMC2408822

[31] Fayard E, Schoonjans K, Annicotte JS, Auwerx J (2003) Liver receptor homolog 1 controls the expression of carboxyl ester lipase. J Biol Chem 278: 35725–35731. 12853459 10.1074/jbc.M302370200

[32] Coste A, Dubuquoy L, Barnouin R, Annicotte JS, Magnier B, et al (2007) LRH-1-mediated glucocorticoid synthesis in enterocytes protects against inflammatory bowel disease. Proc Natl Acad Sci U S A 104: 13098–13103. 17670946 10.1073/pnas.0702440104 PMC1941823

[33] Srinivas S, Watanabe T, Lin CS, William CM, Tanabe Y, et al (2001) Cre reporter strains produced by targeted insertion of EYFP and ECFP into the ROSA26 locus. BMC Dev Biol 1: 4. 11299042 10.1186/1471-213X-1-4 PMC31338

[34] Wen S, Schwarz JR, Niculescu D, Dinu C, Bauer CK, et al (2008) Functional characterization of genetically labeled gonadotropes. Endocrinology 149: 2701–2711. 18325995 10.1210/en.2007-1502

[35] Livak KJ, Schmittgen TD (2001) Analysis of relative gene expression data using real-time quantitative PCR and the 2(-Delta Delta C(T)) Method. Methods 25: 402–408. 11846609 10.1006/meth.2001.1262

[36] Galarneau L, Pare JF, Allard D, Hamel D, Levesque L, et al (1996) The alpha1-fetoprotein locus is activated by a nuclear receptor of the Drosophila FTZ-F1 family. Mol Cell Biol 16: 3853–3865. 8668203 10.1128/mcb.16.7.3853 PMC231382

[37] Dorn C, Ou Q, Svaren J, Crawford PA, Sadovsky Y (1999) Activation of luteinizing hormone beta gene by gonadotropin-releasing hormone requires the synergy of early growth response-1 and steroidogenic factor-1. J Biol Chem 274: 13870–13876. 10318795 10.1074/jbc.274.20.13870

[38] Halvorson LM, Ito M, Jameson JL, Chin WW (1998) Steroidogenic factor-1 and early growth response protein 1 act through two composite DNA binding sites to regulate luteinizing hormone beta-subunit gene expression. J Biol Chem 273: 14712–14720. 9614069 10.1074/jbc.273.24.14712

[39] Kaiser UB, Halvorson LM, Chen MT (2000) Sp1, steroidogenic factor 1 (SF-1), and early growth response protein 1 (egr-1) binding sites form a tripartite gonadotropin-releasing hormone response element in the rat luteinizing hormone-beta gene promoter: an integral role for SF-1. Mol Endocrinol 14: 1235–1245. 10935547 10.1210/mend.14.8.0507

[40] Tremblay JJ, Drouin J (1999) Egr-1 is a downstream effector of GnRH and synergizes by direct interaction with Ptx1 and SF-1 to enhance luteinizing hormone beta gene transcription. Mol Cell Biol 19: 2567–2576. 10082522 10.1128/mcb.19.4.2567 PMC84049

[41] Wen S, Ai W, Alim Z, Boehm U (2010) Embryonic gonadotropin-releasing hormone signaling is necessary for maturation of the male reproductive axis. Proc Natl Acad Sci U S A 107: 16372–16377. 20805495 10.1073/pnas.1000423107 PMC2941299

[42] Lazarus KA, Wijayakumara D, Chand AL, Simpson ER, Clyne CD (2012) Therapeutic potential of Liver Receptor Homolog-1 modulators. J Steroid Biochem Mol Biol 130: 138–146. 22266285 10.1016/j.jsbmb.2011.12.017

[43] Quirk CC, Seachrist DD, Nilson JH (2003) Embryonic expression of the luteinizing hormone beta gene appears to be coupled to the transient appearance of p8, a high mobility group-related transcription factor. J Biol Chem 278: 1680–1685. 12429736 10.1074/jbc.M209906200

[44] Tremblay JJ, Lanctot C, Drouin J (1998) The pan-pituitary activator of transcription, Ptx1 (pituitary homeobox 1), acts in synergy with SF-1 and Pit1 and is an upstream regulator of the Lim-homeodomain gene Lim3/Lhx3. Mol Endocrinol 12: 428–441. 9514159 10.1210/mend.12.3.0073

[45] Tran S, Zhou X, Lafleur C, Calderon MJ, Ellsworth BS, et al.. (2013) Impaired Fertility and FSH Synthesis in Gonadotrope-Specific Foxl2 Knockout Mice. Mol Endocrinol. doi: 10.1210/me.2012–1286. 10.1210/me.2012-1286 PMC3589670 23340250

