# Supplementary figures and images for: NR5A2 Regulates Lhb and Fshb Transcription in Gonadotrope-Like Cells In Vitro, but Is Dispensable for Gonadotropin Synthesis and Fertility In Vivo
Source: PLoS One. 2013 Mar 11;8(3):e59058. doi: 10.1371/journal.pone.0059058 (PMC3594184; doi:10.1371/journal.pone.0059058)

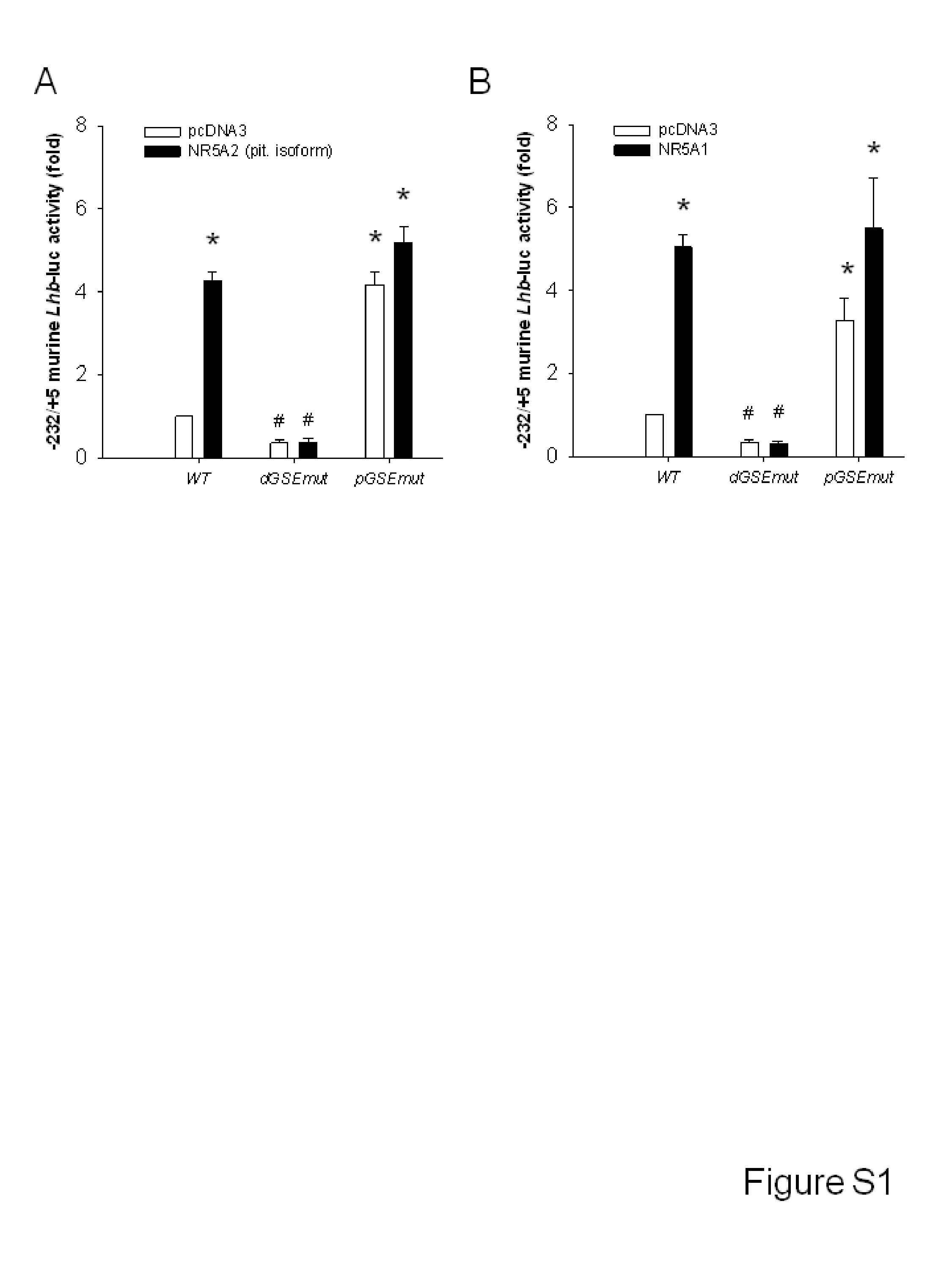

Supplement: Figure S1 — Both conserved GSE elements mediate NR5A2 activation of the murine Lhb promoter. HeLa cells were transfected with 225 ng/well of the murine −232/+5 Lhb-luc reporter or the same reporter carrying a mutation in the proximal (pGSEmut) or distal (dGSEmut) GSE elements along with 50 ng/well of A) NR5A2 (pituitary isoform) or B) NR5A1 expression vectors (black bars) or empty vector (pcDNA3 – white bars). Bars with different symbols differ significantly. Data represent the mean +SEM of five (A) and four (B) independent experiments performed in triplicate. Note that these data are from the same experiments as those shown in Figure 3. Therefore, the values for the wild-type (WT) reporter are the same in both figures. (TIF) [file pone.0059058.s001.tif]

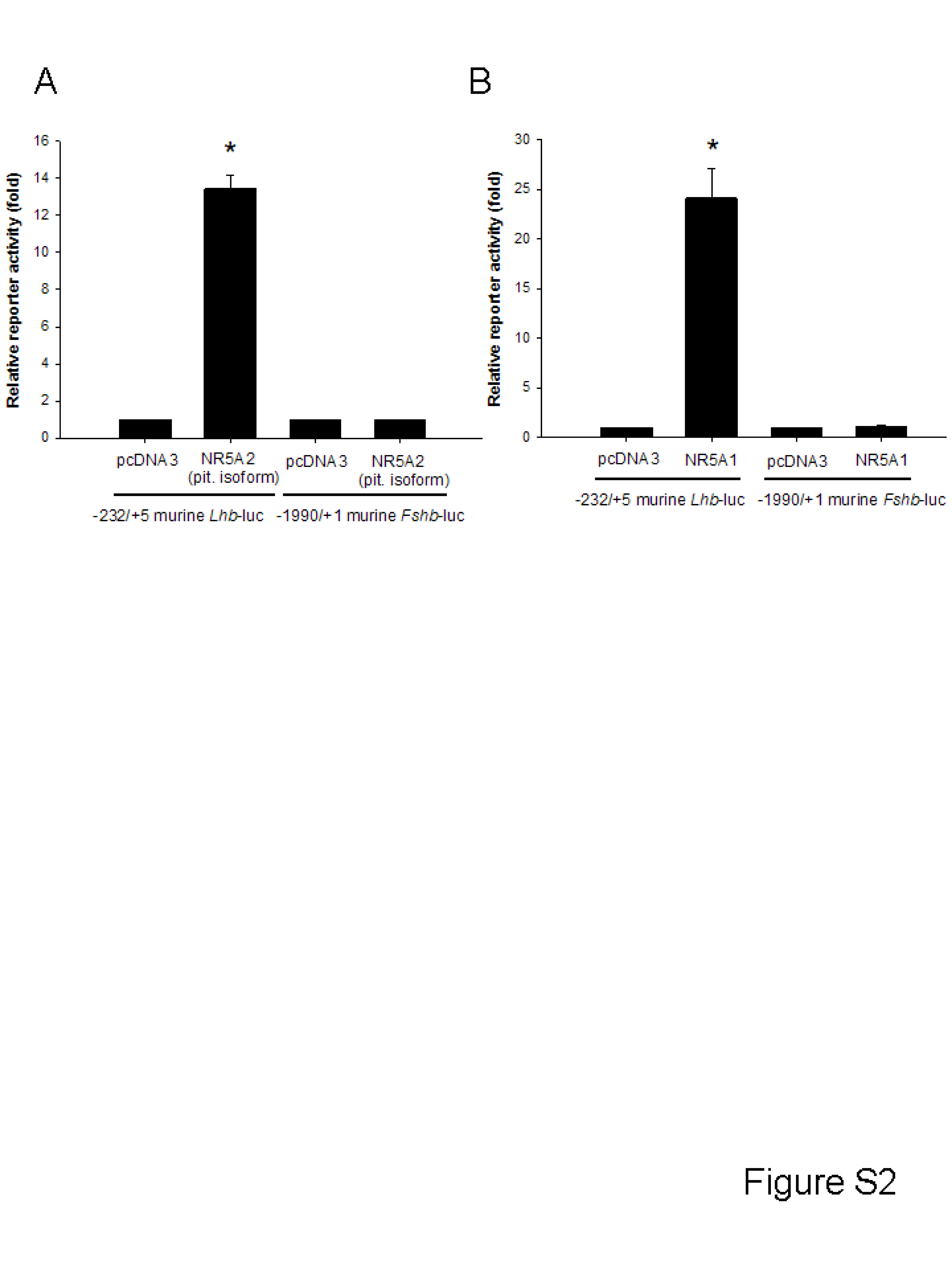

Supplement: Figure S2 — NR5A1 and NR5A2 do not directly activate the murine Fshb promoter. HeLa cells were transfected with 225 ng/well of the murine −232/+5 Lhb-luc or −1990/+1 Fshb-luc reporter as well as 50 ng/well of A) NR5A2 or B) NR5A1 (pituitary isoform) expression vectors or empty vector (pcDNA3). Bars with different symbols differ significantly. Data represent the mean + SEM of three independent experiments performed in triplicate. (TIF) [file pone.0059058.s002.tif]

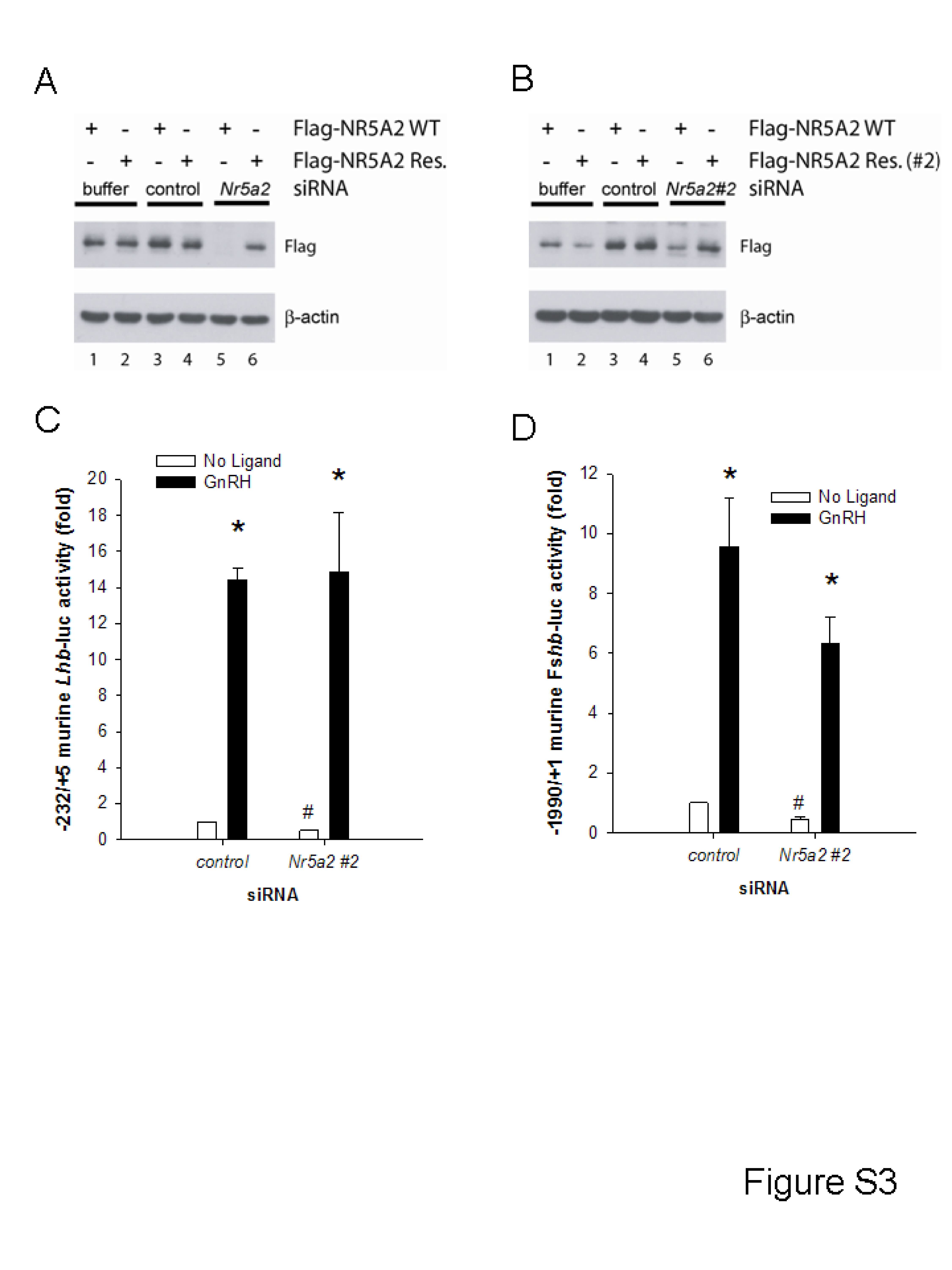

Supplement: Figure S3 — Validation of the Nr5a2 siRNAs used in this study. A) and B) CHO cells were transfected with wild-type (WT) or siRNA-resistant (Res.) forms of Flag-tagged NR5A2 along with 5×10^−9^ M non-specific (control) or Nr5a2 siRNAs, or 1X siRNA dilution buffer. Whole-cell lysates were collected and subjected to anti-Flag (top panel) or anti-β-actin (bottom panel) western blot analyses. C) and D) LβT2 cells were transfected with 225 ng/well C) murine −232/+5 Lhb-luc or D) murine −1990/+1 Fshb-luc reporters. Cells were co-transfected with Nr5a2 siRNA #2, or a non-specific siRNA (control) at a final concentration of 5×10^−9^ M. Cells were treated with 10−7 M GnRH for 6 h (black bars) or left untreated (white bars) prior to collection of whole cell lysates for luciferase assays. Bars with different symbols differ significantly. Data represent the mean +SEM of three (C), or seven (D) independent experiments performed in triplicate. The data presented in panels C and D of this figure are from the same experiments as those of Figure 4A and 4C , respectively. Therefore, the values for the “control” siRNA condition in the two figures are the same. (TIF) [file pone.0059058.s003.tif]

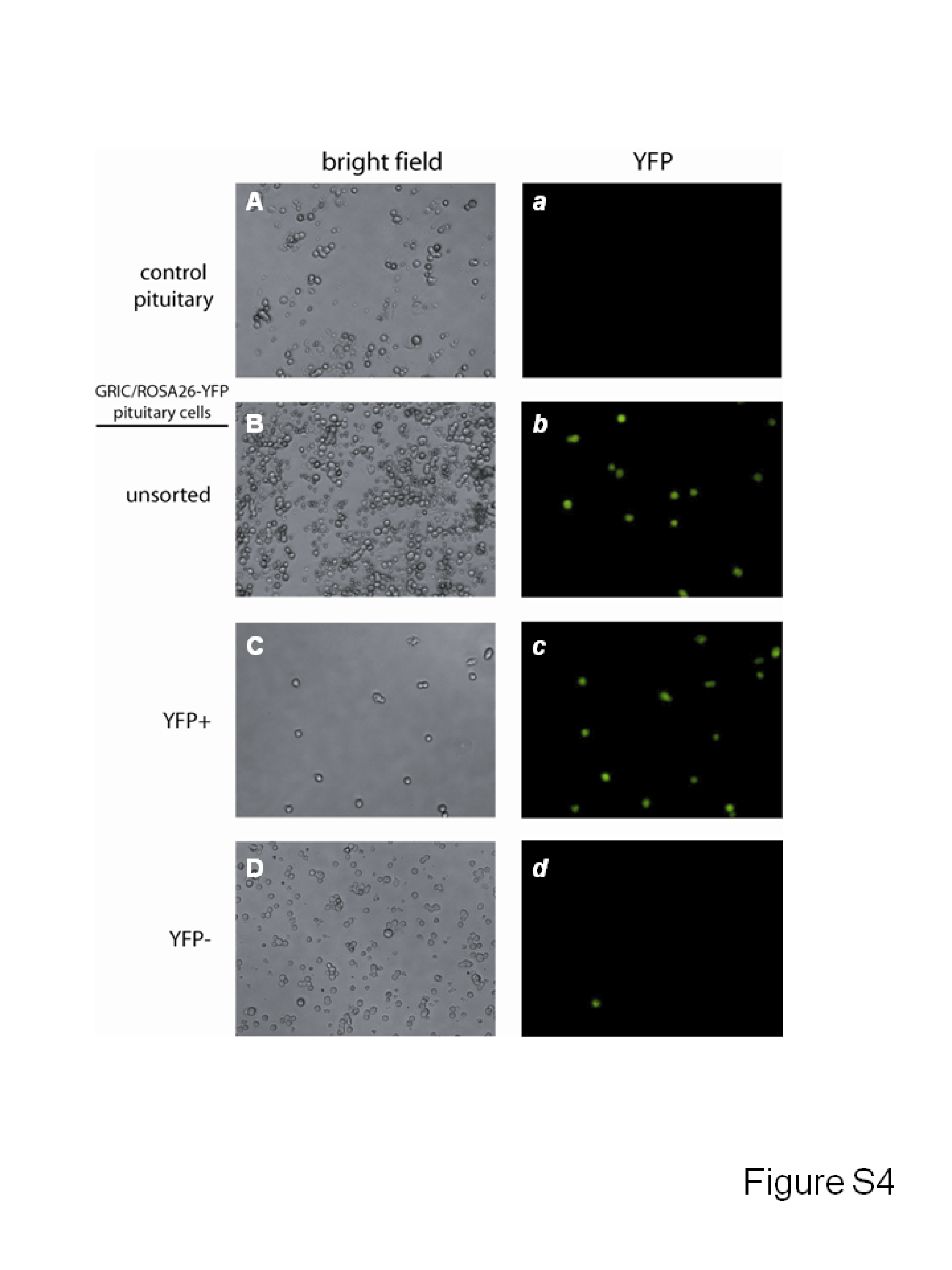

Supplement: Figure S4 — Efficient and selective purification of YFP + gonadotropes from GnrhrGRIC /+ ; ROSA26eYFP /+ mice. Dissociated pituitary cells from wild-type (A, a) and GnrhrGRIC/+;ROSA26eYFP/+ mice (B, b–D, d) were plated in primary culture. A–D) Pictures of cells taken under brightfield illumination. a–d) The same cells as in A–D, but viewed under fluorescent illumination for detection of YFP. B, b) Cells from GnrhrGRIC/+; ROSA26eYFP/+ mice prior to FACS. C, c) Cells from the YFP + fraction following FACS. D, d) Cells from the YFP- fraction following FACS. A field with a single YFP + cell is shown, but most fields examined lacked YFP + cells. (TIF) [file pone.0059058.s004.tif]

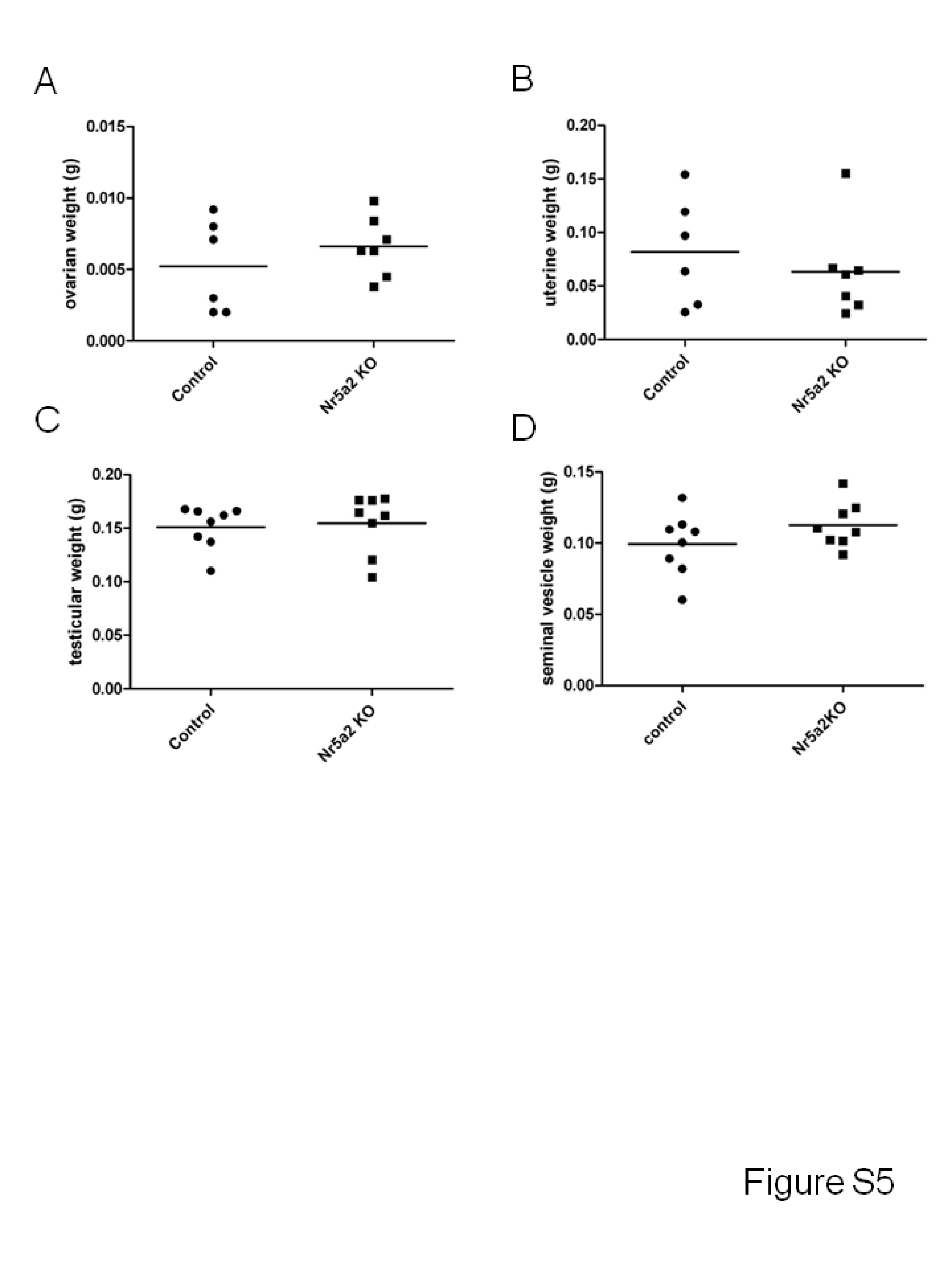

Supplement: Figure S5 — Reproductive organ weights in gonadotrope-specific Nr5a2 knockout mice. Ovarian (A) and uterine (B) weights were measured in 6 week-old female Nr5a2fl/fl (control, n = 6) and Nr5a2fl/fl;GnrhrGRIC/+ (Nr5a2KO, n = 6) mice. Testicular (C) and seminal vesicle (D) weights were measured in 6 week-old male mice (control, n = 8; Nr5a2KO, n = 8, bars = means). (TIF) [file pone.0059058.s005.tif]

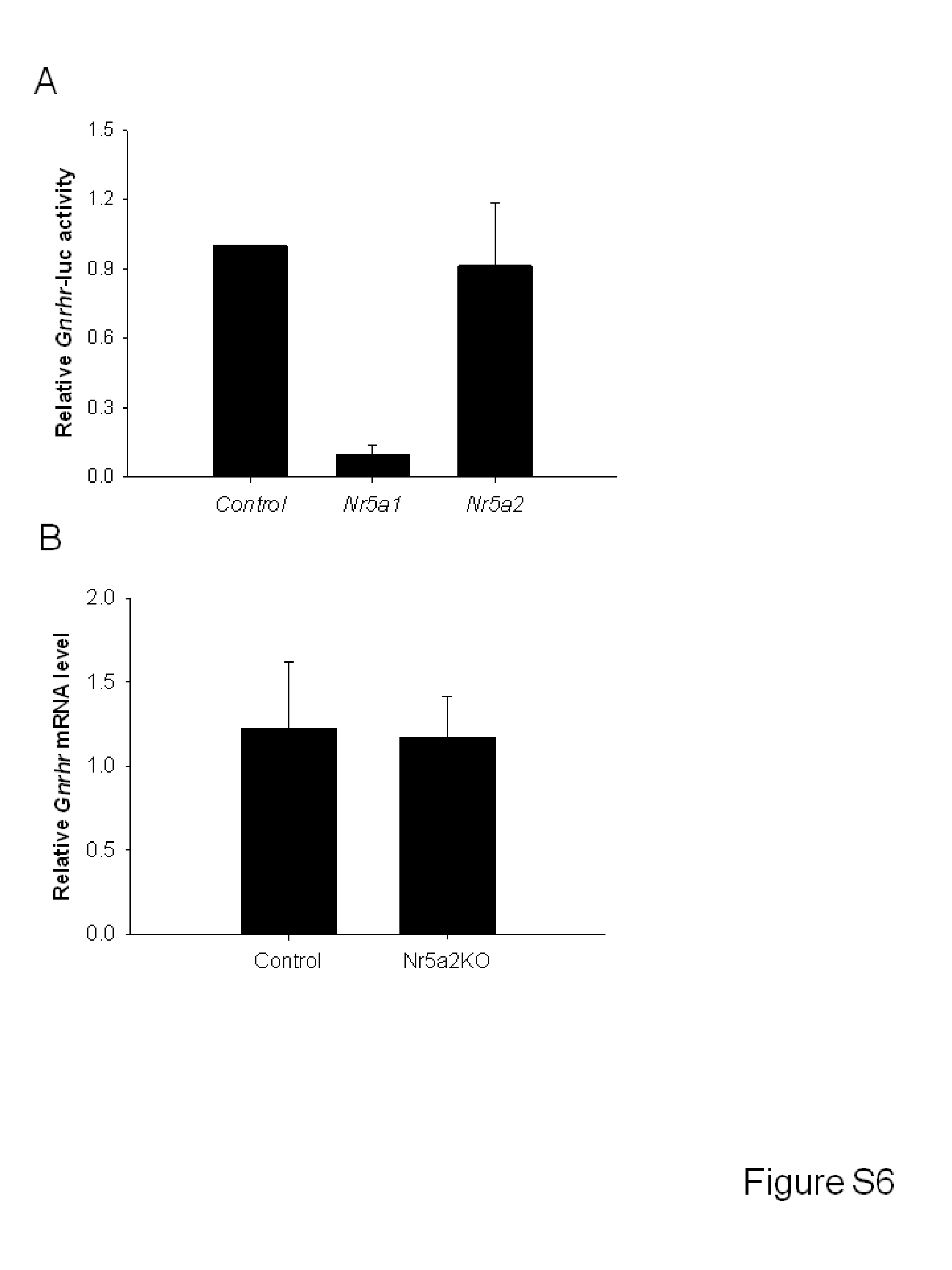

Supplement: Figure S6 — Nr5a2 does not regulate gonadotrope expression of Gnrhr in vitro or in vivo . A) LβT2 cells were transfected with 225 ng/well of the −1772/+38 murine Gnrhr-luc reporter. Cells were co-transfected with control, Nr5a1 or Nr5a2 siRNAs as indicated, at a final concentration of 5×10^−9^ M. Data represent the mean + SEM of four independent experiments performed in triplicate. B) cDNA was prepared from total RNA isolated from individual pituitary glands of Nr5a2fl/fl (control, n = 5) and Nr5a2fl/fl;GnrhrGRIC/+ (Nr5a2KO, n = 8) male mice, and analyzed for expression of Gnrhr by quantitative real-time PCR. (TIF) [file pone.0059058.s006.tif]

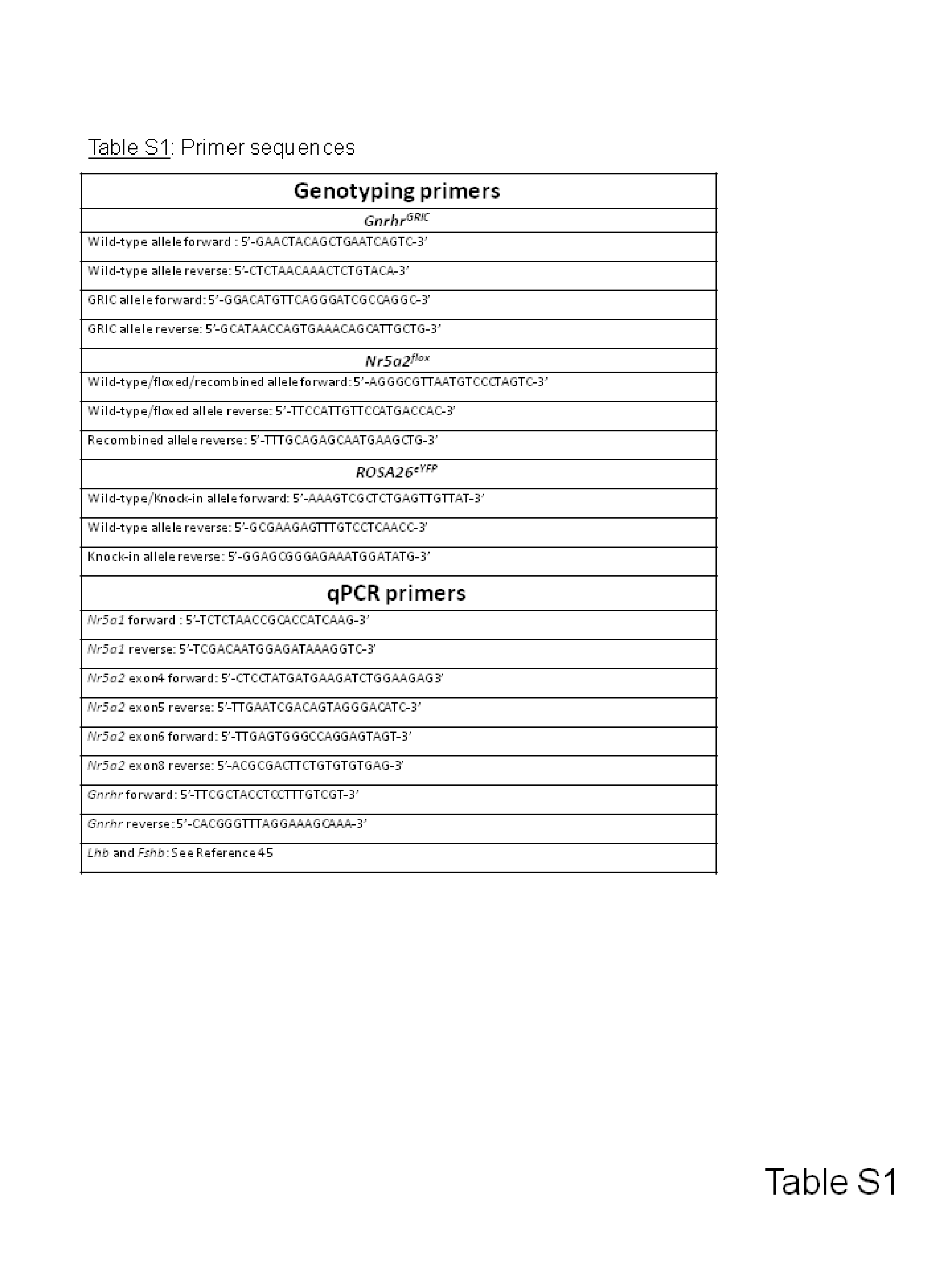

Supplement: Table S1 — Primer sequences. (TIF) [file pone.0059058.s007.tif]
